# No differences in functional and clinical outcomes after rehabilitation between modified kinematic and mechanical alignment in total knee arthroplasty: A randomized controlled trial

**DOI:** 10.1002/ksa.70004

**Published:** 2025-08-19

**Authors:** Leandra Bauer, Frank Layher, Julia Kirschberg, Markus Heinecke, Matthias Woiczinski, Georg Matziolis

**Affiliations:** ^1^ Experimental Orthopaedics, University Hospital Jena, Campus Eisenberg, Waldkliniken Eisenberg Friedrich‐Schiller‐University Jena Jena Germany; ^2^ Orthopaedics, University Hospital Jena, Campus Eisenberg, Waldkliniken Eisenberg Friedrich‐Schiller‐University Jena Jena Germany

**Keywords:** alignment techniques, gait analysis, knee kinematics, patient‐reported outcome measure, total knee arthroplasty

## Abstract

**Purpose:**

Despite advancements in total knee arthroplasty (TKA), patient dissatisfaction remains notably high (15%–25%). This dissatisfaction will be multifactorial, one of which may be the alignment of the components. Kinematic alignment (KA), aimed at restoring pre‐arthritic knee anatomy, is proposed as a promising alternative to mechanical alignment (MA), potentially offering better functional outcomes and improved gait characteristics.

**Methods:**

A randomized controlled trial was conducted involving 100 patients undergoing primary TKA. Patients were randomized into two groups (KA vs. MA) using a navigation‐assisted surgical approach, with follow‐ups conducted at 1 year post‐operatively. Outcomes assessed included patient‐reported outcome measures (Knee Society Score, Western Ontario and McMaster Universities Osteoarthritis Index, Visual Analogue Scale and Forgotten Joint Score), radiological alignment and comprehensive gait analysis (kinematics, kinetics and spatio‐temporal parameters).

**Results:**

Navigation analyses indicated significant post‐operative alignment differences, with KA showing significantly more varus tibial (KA: 2.2 ± 2.8° vs. MA: 0.3 ± 0.6°, *p* < 0.001) and more valgus femoral cuts (KA: −0.7 ± 2.9° vs. MA: 0.3 ± 0.8°, *p* = 0.02) compared to MA. However, these differences did not translate into significant clinical or functional differences between groups in patient‐related outcome measures, gait kinematics, kinetics, or spatio‐temporal parameters at the 1‐year mark. Both alignment techniques showed similar deviations from healthy gait patterns, particularly reduced knee flexion (mean ROM healthy 57.3°, KA 48.6°, MA 47.8°), and knee valgus during walking (mean maximal valgus healthy 15.2°, KA 10.5°, MA 9.5°). Notably, KA required fewer intraoperative soft tissue releases, suggesting procedural simplicity.

**Conclusion:**

This study found no significant differences in clinical or functional outcomes between KA and MA despite distinct radiological alignment outcomes after 1‐year follow‐up. Both approaches yield comparable patient satisfaction and functional performance 1 year post‐operatively. KA offers procedural advantages, specifically reduced soft tissue interventions.

**Level of Evidence:**

Level I.

**Abbreviations**
FJS: Forgotten Joint Score
GRF: ground reaction force
HKA: hip–knee–ankle
KA: kinematic alignment
KSS: Knee Society Score
MA: mechanical alignment
PROM: patient‐related outcome measure
SPM: statistical parametric mapping
TKA: total knee arthroplasty
VAS: visual analogue scale
WOMAC: Western Ontario and McMaster Universities Osteoarthritis Index

## INTRODUCTION

In contrast to hip arthroplasty, the clinical outcomes following total knee arthroplasty (TKA) remain unsatisfactory, with 15%–25% of patients reporting persistent dissatisfaction [9, 33]. In Germany, approximately 156,000 knee arthroplasties are performed annually [13], meaning that over 35,000 patients each year remain permanently dissatisfied with their surgical outcomes. It is well‐established that this dissatisfaction arises from various factors [17, 29]. The fact that only 5% of patients report dissatisfaction following hip replacement—despite comparable indications, preparation and post‐operative care for hip and knee arthroplasty—suggests that the causes lie within the surgery itself (e.g., implant design, positioning and surgical technique) or within the more complex joint complexity due to different anatomical coupling mechanism within the hip or knee.

To date, no prosthesis has been able to reliably restore the complex kinematics of the natural knee joint [5, 14] as reflected by pathological gait patterns [25] and suboptimal ‘forgotten knee scores’ [2, 36]. Even the implant geometries of competing design philosophies (e.g., single‐radius vs. J‐curve) differ by only a few millimetres—an order of variation in intraoperative component positioning, which in turn limits the potential of modern designs [28].

Alignment strategies markedly influence post‐operative function. Kinematic alignment (KA) aims to restore the pre‐arthritic anatomy of the knee and has emerged as a promising alternative to the traditionally used mechanical alignment (MA) [16]. Several studies have demonstrated that KA better replicates physiological gait patterns compared to MA, resulting in improved symmetry, knee kinematics, and more natural walking characteristics [3, 21], but in some cases with a lack of clinical significance [32]. In particular, Blakeney et al. highlighted that patients undergoing KA show significantly more physiological gait mechanics than those undergoing MA, characterized by increased knee flexion and more symmetrical gait patterns. Furthermore, KA more faithfully reproduces native patellar tracking patterns [18] and trochlear anatomy, with lower rates of patellofemoral complications [22]. However, MRI assessments question the universal application of a 2 mm tolerance in KA, revealing substantial inter‐individual variation in femoral cartilage thickness [12].

Clinical evidence for KA remains heterogeneous, in part due to inconsistent definitions and reporting. Umbrella reviews and meta‐analyses often group disparate KA techniques and fail to standardize adverse‐event reporting, rendering comparisons of complication and revision rates unreliable [6]; primary studies likewise seldom report key alignment parameters such as medial proximal tibial angle, lateral distal femoral angle and hip–knee–ankle (HKA) in detail [26]. Nevertheless, selected randomized trials and meta‐analyses have reported improved functional scores and higher patient satisfaction with KA [4, 8, 10, 30], although follow‐up durations are generally short and outcome measures vary widely.

Nevertheless, studies have highlighted limitations, including retrospective designs, short follow‐up durations, and variability in clinical results. Tuecking et al. underscored the ongoing debate regarding the impact of KA on implant survival, although recent evidence suggests that individualized alignment strategies do not negatively impact implant longevity [31]. Furthermore, Yeo et al. reported no significant clinical or gait differences between robotic‐assisted MA and anatomical alignment [34], underlining the complexity of translating biomechanical advantages into clear clinical benefits.

Accordingly, there remains a clear need for high‐quality, prospective, randomized studies with comprehensive post‐operative rehabilitation. Our research group has developed a modified KA technique in which the femoral component is first aligned parallel to the transepicondylar axis under navigation, followed by sequential femoral and tibial gap balancing to achieve symmetrical extension and flexion gaps [20]. Building on promising pilot data, the present study compares clinical and functional outcomes of this modified KA approach with a traditional gap‐technique MA and healthy controls after 1 year of rehabilitation. We hypothesize that the modified KA technique will yield superior gait parameters and patient‐reported outcome measures.

## MATERIALS AND METHODS

The study was approved by the local ethics committee (IRB no. 5060‐02/17), and all participants signed an informed consent. All methods were performed in accordance with the relevant guidelines and regulations.

### Patients and randomization

A single‐centre single‐blinded randomized controlled trial was conducted. A power analysis was performed using G*Power 3.1 based on the Mann–Whitney *U* test for the primary outcome variable FJS‐12, assuming a clinically relevant difference of 10 points between groups (conventional: mean = 75, SD = 15; new technique: mean = 85, SD = 17), *α* = 0.05 and power = 0.80. This resulted in a required sample size of at least 35 patients per group, and thus, 50 patients per group were included to account for potential dropouts and lost‐to‐follow‐up cases. Therefore, a total of 100 patients with an indication for primary TKA were initially included in this study.

Inclusion criteria were the indication for implantation of a primary TKA and an age of over 18 years. Exclusion criteria were post‐traumatic osteoarthritis of the affected joint, previous operations with the exception of arthroscopic operations, TKA on the contralateral side, total hip arthroplasty regardless of the side, spinal disease leading to a reduced walking distance or a clinically perceptible pathological gait pattern (e.g. spinal canal stenosis and degenerative lumbar scoliosis), underlying neurological disease affecting the gait pattern, history of depression.

The patients were randomly assigned to one of the two study groups immediately before the operation and remained blinded until the end of the study—one undergoing KA and the other MA. Randomization was conducted using a random number generator, employing block randomization with a fixed block size of four. Within each block, two participants were randomly assigned to each intervention group, ensuring balanced group sizes throughout the recruitment period.

The modified KA was performed by using the transspherical axis to align the femoral component. All surgeries were performed using a navigation system (OrthoPilot, Aesculap AG, B. Braun). Study nurses and staff who conducted the clinical follow‐up were also blinded to group allocation. The surgeon, who was not blinded, analyzed the X‐ray images.

Since all surgeries were performed using navigation, the following parameters could be determined based on the navigation protocols: Preoperative alignment of the femur, tibia, and overall mechanical axis; Positioning of the implants in relation to the mechanical axes; Absolute height of the flexion and extension gaps, medially and laterally; Soft tissue release: performed (yes/no), and if yes, the effect on the gap dimensions.

All patients received pre‐operative assessment as well as 1 year after surgery.

### Gait analysis and patient‐related outcome measures (PROMs)

The assessment included PROMs with Knee Society Score (KSS), Western Ontario and McMaster Universities Osteoarthritis Index (WOMAC) and visual analogue scale (VAS) to quantify pain frequency and strength, preoperatively and at 12 months post‐operatively. Forgotten Joint Score (FJS) was assessed only at 12 months post‐operatively. The FJS is a validated patient‐reported outcome measure, which evaluates the patients' ability to ‘forget’ the artificial joint in daily activities, and has demonstrated high internal consistency (Cronbach's *ɑ* = 0.95) and discriminative validity, with lower ceiling effects compared to traditional measures such as the WOMAC index [1].

Furthermore, all patients received an instrumented 3D gait analysis. Kinematic data were acquired using an optical motion capture system with 10 infrared cameras (Bonita 10, Vicon Motion Systems, sample rate 200 Hz) with the Plug‐In‐Gait marker set for the lower extremity. Patients were instructed to walk along a 5‐m walkway six times at their preferred, comfortable walking speed. The gait speed was measured with permanently installed light barriers at the beginning and end of the walkway. The walkway was equipped with three embedded force plates to record ground reaction forces (GRFs)—one Kistler device (Kistler Instruments AG), and two AMTI devices (AMTI). Data from 12 steps (6 per side) around the centre of the force plate were analyzed for each patient using Nexus V.2.1 and Polygon V.4.1 (Vicon Motion Systems).

### Data analysis and statistics

All further data analysis was carried out by a self‐made script using MATLAB (Version 24.2, 2024b, The MathWorks Inc.).

In order to quantify the change in the PROMS, the delta score was calculated in terms of post‐op and pre‐op. The gait data were normalized to 100% of the gait cycle in 1% increments, and the kinetic data were normalized to body weight. The mean value and the standard deviation (SD) were then calculated for each patient for the 12 steps included for the affected and unaffected sides. The mean and SD were subsequently calculated for all data for each MA and KA group. The results were compared to a reference dataset of healthy individuals (*n* = 50, 25F/25M, age 61.2 ± 6.1 years, body mass index [BMI] 26.7 ± 4.1) without any knee joint discomforts.

To compare time‐series data from KA and MA groups, post‐op statistical parametric mapping (SPM) for unpaired *t* test (*p* = 0.05) was carried out [23].

For parameters with pre‐/post‐op values (spatio‐temporal parameters from gait analysis, PROMs and HKA angle), a 2 × 2 mixed factorial analysis of variance (ANOVA) was performed with SPSS (IBM Corp. Released 2024, IBM SPSS Statistics for MacOS, Version 30.0.0). For each parameter, a repeated‐measures ANOVA was specified with Time (two levels: Pre vs. Post) as the within‐subjects factor and Group (two levels: MA vs. KA) as the between‐subjects factor. Greenhouse–Geisser corrections were applied when Mauchly's test indicated violations of sphericity. Significant main effects and interactions were followed up with Bonferroni‐adjusted pairwise comparisons to identify specific differences between time points within each surgical group. For parameters with only post‐op values (FJS, femoral and tibial cut), an unpaired *t* test was performed for an independent group comparison (MA vs. KA). Statistical significance was set at *α* = 0.05 for all tests.

## RESULTS

### Demographic patient data

**Figure 1:**
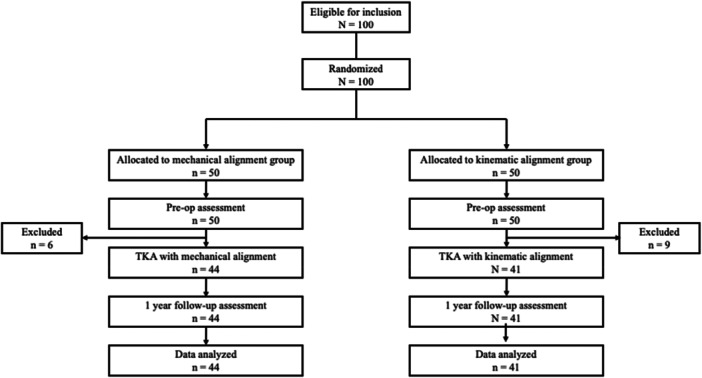
Flow‐chart of patient enrolment process. op, operative, TKA, total knee arthroplasty.

Of the initially enroled 100 patients, 15 were lost to follow‐up, resulting in final group sizes of *n* = 44 for MA and *n* = 41 for KA (see Figure 1). There were no significant differences between the groups in terms of height, weight or BMI either preoperatively or post‐operatively (*p* > 0.05, see Table 1).

**Table 1:** Demographic patient data for mechanical alignment (MA) and kinematic alignment (KA) groups with mean ± standard deviation (SD).

Parameter	Group	Time point	M ± SD	*F*_time_ (*p*)	*F*_group_ (*p*)	*F*_int_ (*p*)
Body weight [kg]	MA	Pre‐OP	93.9 ± 14.3	0.18 (0.67)	0.85 (0.36)	0.29 (0.59)
	MA	Post‐OP	95.7 ± 17.1			
	KA	Pre‐OP	92.0 ± 18.7			
	KA	Post‐OP	91.8 ± 17.1			
Body height [m]	MA	Pre‐OP	1.7 ± 0.1	0.57 (0.45)	1.43 (0.24)	0.84 (0.36)
	MA	Post‐OP	1.7 ± 0.1			
	KA	Pre‐OP	1.7 ± 0.1			
	KA	Post‐OP	1.7 ± 0.1			
BMI [kg/m^2^]	MA	Pre‐OP	32.7 ± 5.1	0.00 (0.99)	0.03 (0.86)	1.06 (0.31)
	MA	Post‐OP	33.3 ± 5.8			
	KA	Pre‐OP	33.1 ± 6.4			
	KA	Post‐OP	32.5 ± 6.3			

*Note*: *F* and *p* value for time, group and int = time × group.

Abbreviations: BMI, body mass index; OP, operative.

### Clinical outcome: HKA

In the clinical data, the groups did not differ significantly in the preoperative HKA angle (*p *= 0.211). Following surgery, the HKA angle was corrected to 0.2 ± 1.1° in the MA group, whereas the KA group resulted in an HKA angle of 1.4 ± 1.9°. This improvement over time was highly significant in both groups (pre‐ vs. post‐operative contrast: *p* < 0.001 for MA and *p* < 0.001 for KA), while neither the between‐group difference at endpoint nor the group × time interaction reached significance (group: *p* = 0.211; interaction: *p* = 0.512) The femoral cut angle was significantly different between groups (*p* = 0.02), with the MA group showing slightly more valgus. Likewise, the tibial cut angle differed significantly, being more varus in the KA group (*p* < 0.001; see Table 2).

**Table 2:** Clinical data from navigation protocol—Hip–knee–ankle (HKA) angle and femoral/tibial cut for mechanical alignment (MA) and kinematic alignment (KA) groups.

Parameter	Group	Time point	Mean ± SD	*F*_time_ (*p*)		*F*_group_ (*p*)		*F*_int_ (*p*)	Pre vs. post, *p* (post hoc)
HKA [°]	MA	Pre‐OP	4.2 ± 5.7	60.26 (<0.001^a^)	1.59 (0.21)		0.43 (0.51)		<0.001^a^
	MA	Post‐OP	0.2 ± 1.1						
	KA	Pre‐OP	5.0 ± 4.7						<0.001^a^
	KA	Post‐OP	1.4 ± 1.9						

Parameter	Group	Time point	Mean ± SD	*T* (*p*)					
Femoral cut (+varus) [°]	MA	Intra‐OP	0.3 ± 0.8	2.12 (0.02^b^)					
	KA	Intra‐OP	−0.7 ± 2.9						
Tibial cut (+varus) [°]	MA	Intra‐OP	0.3 ± 0.6	−4.48 (<0.001^b^)					
	KA	Intra‐OP	2.2 ± 2.8						

*Note*: *F* and *p* value for time, group and int = time × group, *T* and *p* value for unpaired *t* test.

Abbreviations: OP, operative; SD, standard deviation.

a Time effect is significant at *α* = 0.05. The pairwise contrasts pre versus post are highly significant for both groups (Bonferroni‐corrected *p* < 0.001).

^b^ Group effect for MA versus KA is significant at *α* = 0.05.

### PROMs

In the PROMs, both groups experienced significant improvements over time in knee‐specific scores and overall function, without any between‐group differences or interactions. Specifically, the KSS for knee evaluation increased markedly from 44.9 ± 15.6 to 84.7 ± 12.0 in the MA group and from 40.8 ± 18.0 to 81.2 ± 14.1 in the KA group (Time effect: *F*(1,83) = 276.4, *p* < 0.001; both pre‐ vs. post‐op contrasts *p* < 0.001). Likewise, the KSS functional evaluation rose from 60.5 ± 16.9 to 78.4 ± 15.3 for MA and from 63.5 ± 16.0 to 83.3 ± 11.7 for KA (*F*(1,83) = 79.47, *p* < 0.001; both contrasts *p* < 0.001). WOMAC scores similarly showed a robust time effect (from 50.8 ± 21.5 to 15.2 ± 16.9 in MA and 45.9 ± 24.3 to 15.4 ± 19.0 in KA; *F*(1,83) = 185.86, *p* < 0.001; Group: *p* = 0.55; Interaction: *p* = 0.30; both contrasts *p* < 0.001). In contrast, pain frequency (VAS frequency) and pain intensity (VAS strength) remained stable across time with no Group or Interaction effects (all *p* > 0.27). At 12 months, the FJS did not differ between groups (*p* = 0.42; see Table 3).

**Table 3:** Mean ± standard deviation (SD) for patient‐related outcome measurements (KSS—Knee Society Score, WOMAC—Western Ontario and McMaster Universities Osteoarthritis Index, VAS—visual analogue scale for frequency and strength, FJS—Forgotten Joint Score) for pre‐ and post‐operative for mechanical alignment (MA) and kinematic alignment (KA) groups.

Parameter	Group	Time point	Mean ± SD	*F*_time_ (*p*)	*F*_group_ (*p*)	*F*_int_ (*p*)	Pre vs. post, *p* (post hoc)
KSS: knee evaluation	MA	Pre‐OP	44.9 ± 15.6	276.4 (<0.001^a^)	2.83 (0.096)	0.022 (0.88)	<0.001^a^
	MA	Post‐OP	84.7 ± 12.0				
	KA	Pre‐OP	40.8 ± 18.0				<0.001^a^
	KA	Post‐OP	81.2 ± 14.1				
KSS: function evaluation	MA	Pre‐OP	60.5 ± 16.9	79.47 (<0.001^a^)	2.07 (0.15)	0.12 (0.74)	<0.001^a^
	MA	Post‐OP	78.4 ± 15.3				
	KA	Pre‐OP	63.5 ± 16.0				<0.001^a^
	KA	Post‐OP	83.3 ± 11.7				
VAS frequency	MA	Pre‐OP	3.4 ± 3.0	1.49 (0.23)	0.93 (0.34)	0.01 (0.91)	0.23
	MA	Post‐OP	3.7 ± 2.8				
	KA	Pre‐OP	3.9 ± 2.5				0.23
	KA	Post‐OP	4 1. ± 3.0				
VAS strength	MA	Pre‐OP	3.3 ± 2.9	0.16 (0.69)	1.24 (0.27)	0.76 (0.39)	0.27
	MA	Post‐OP	3.7 ± 2.7				
	KA	Pre‐OP	4.1 ± 2.5				0.27
	KA	Post‐OP	3.8 ± 2.6				
WOMAC	MA	Pre‐OP	50.8 ± 21.5	185.86 (<0.001^a^)	0.37 (0.55)	1.09 (0.30)	<0.001^a^
	MA	Post‐OP	15.2 ± 16.9				
	KA	Pre‐OP	45.9 ± 24.3				<0.001^a^
	KA	Post‐OP	15.4 ± 19.0				

Parameter	Group	Time point	Mean ± SD	*T* (*p*)			
FJS	MA	Post‐OP	51.7 ± 24.9	0.81 (0.42)			
	KA	Post‐OP	47.2 ± 26.6				

*Note*: *F* and p value for time, group and int = time × group. *T* and *p* value for unpaired *t* test.

Abbreviation: OP, operative.

a Time effect is significant at *α* = 0.05. The pairwise contrasts pre versus post are highly significant for both groups (Bonferroni‐corrected *p* < 0.001).

Moreover, the distribution and range of values were highly comparable between the two groups. Range, median and distribution are presented by violin plots in Supporting Information S1.

### Gait analysis

Time‐discrete gait analysis variables, including walking speed, stance phase, cadence, and step length, showed no significant differences between the two groups either preoperatively or post‐operatively (*p* > 0.05, see Table 4).

**Table 4:** Spatio‐temporal parameters for gait analysis pre‐ and post‐operative for mechanical alignment (MA) and kinematic alignment (KA) groups.

Parameter	Group	Time point	*M* ± SD	*F*_time_ (*p*)	*F*_group_ (*p*)	*F*_int_ (*p*)
Stance phase [%]	MA	Pre‐OP	63.1 ± 2.4	3.35 (0.71)	0.26 (0.61)	2.55 (0.11)
	MA	Post‐OP	62.2 ± 2.2			
	KA	Pre‐OP	62.5 ± 2.3			
	KA	Post‐OP	62.4 ± 2.6			
Cadence [steps/min]	MA	Pre‐OP	108.8 ± 8.9	3.27 (0.74)	2.62 (0.11)	0.32 (0.58)
	MA	Post‐OP	111.4 ± 9.9			
	KA	Pre‐OP	106.9 ± 7.6			
	KA	Post‐OP	108.3 ± 8.5			
Walking speed [m/s]	MA	Pre‐OP	1.0 ± 0.2	2.87 (0.09)	2.29 (0.13)	0.63 (0.43)
	MA	Post‐OP	1.1 ± 0.2			
	KA	Pre‐OP	1.0 ± 0.2			
	KA	Post‐OP	1.0 ± 0.2			
Step length [m]	MA	Pre‐OP	0.6 ± 0.1	0.76 (0.39)	0.31 (0.58)	0.43 (0.51)
	MA	Post‐OP	0.6 ± 0.1			
	KA	Pre‐OP	0.6 ± 0.1			
	KA	Post‐OP	0.6 ± 0.1			

*Note*: *F* and *p* value for time, group and int = time × group.

Abbreviation: OP, operative.

**Figure 2:**
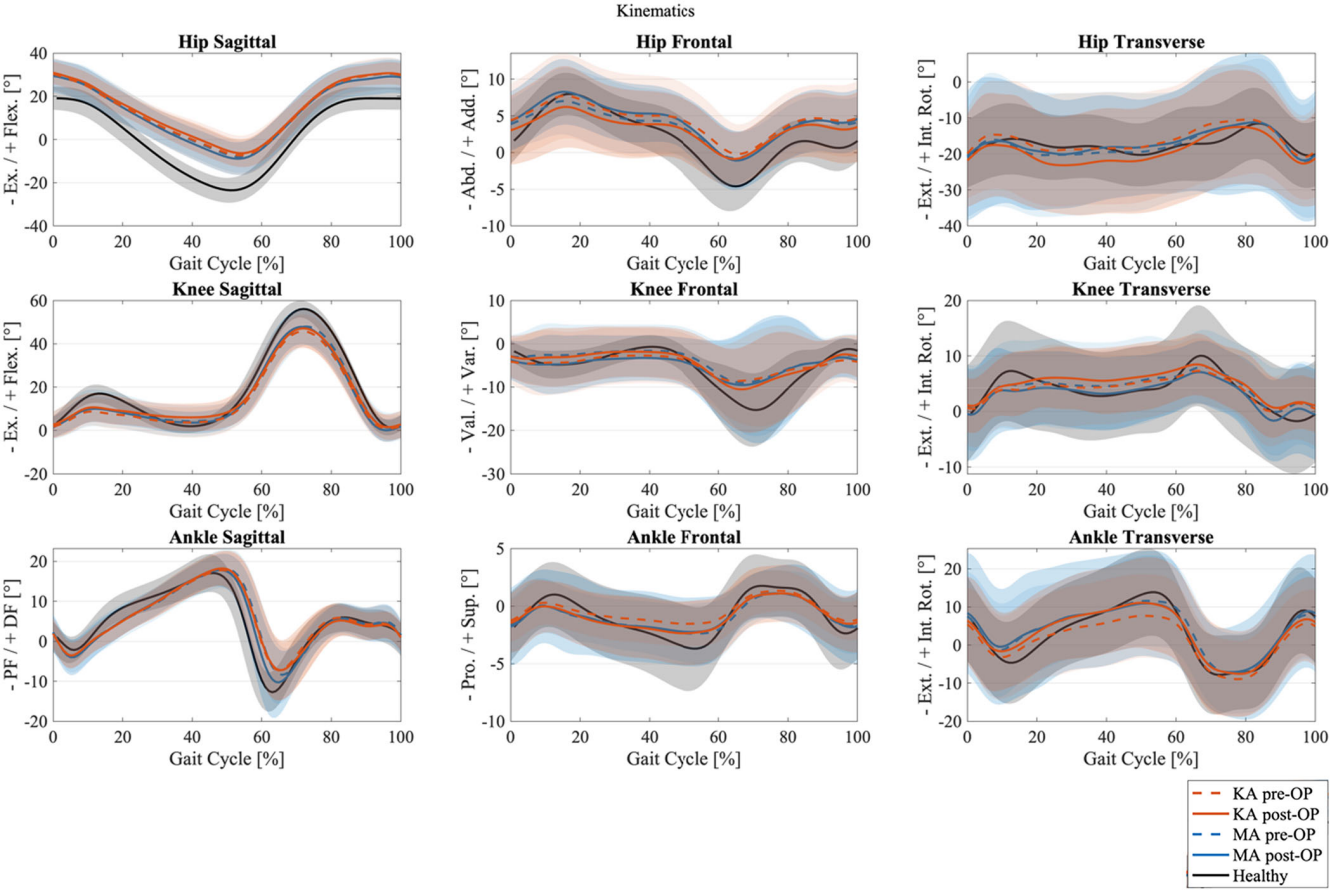
Kinematics for hip, knee and ankle joint in sagittal, frontal and transverse plane for kinematic alignment (orange), mechanical alignment (blue) and healthy controls (black) with mean and standard deviation; dashed line for pre‐OP and solid line for post‐OP. OP, operative.

Mean values and SDs of kinematics are shown in Figure 2 for the hip, knee and ankle joints in the sagittal, frontal and transverse planes. Solid lines represent post‐op and dashed lines pre‐op. The healthy reference cohort is shown in black. The results of the SPM for the post‐operative comparisons are attached in Supporting Information S1.

The kinematic analysis using SPM revealed no significant differences between KA and MA in any of the movement planes. In the sagittal plane, no differences were observed, although hip kinematics exhibited a higher SD. Similarly, in the frontal plane, no significant differences were detected between the two groups, but the overall SD was higher compared to the sagittal plane. In the transverse plane, no significant differences were found either, although MA showed a higher SD. In the sagittal and frontal planes of the knee, deviations from the healthy reference group were observed in both groups 1 year post‐operatively. These deviations were characterized by reduced knee flexion during the load response and mid‐swing phase (mean ROM healthy 57.3°, KA 48.6°, MA 47.8°), as well as reduced knee valgus (mean maximal valgus healthy 15.2°, KA 10.5°, MA 9.5°).

**Figure 3:**
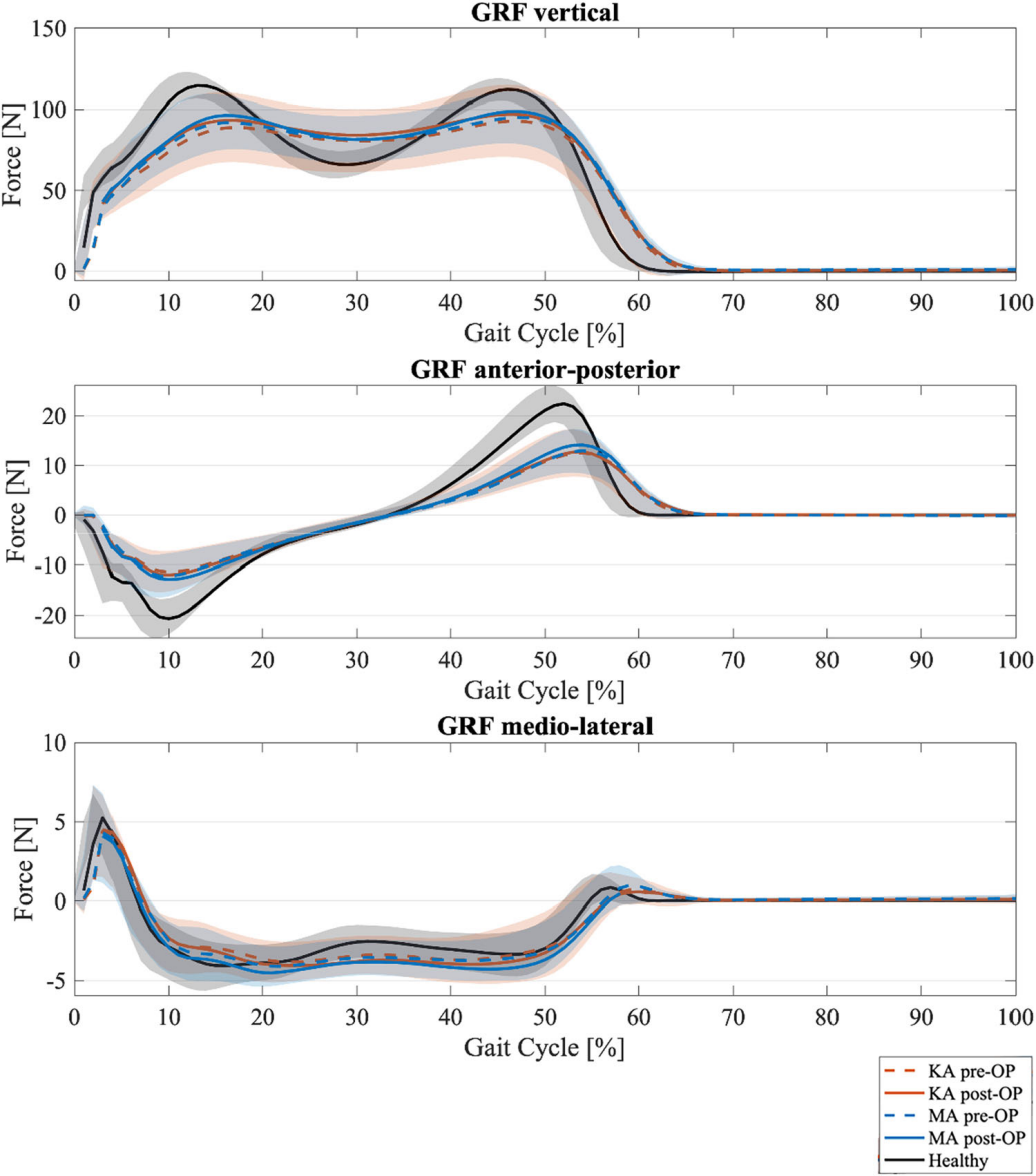
Ground reaction force (GRF) in vertical, horizontal (medio‐lateral) and anterior‐posterior direction for kinematic alignment (orange), mechanical alignment (blue) and healthy controls (black) with mean and standard deviation; dashed line for pre‐OP and solid line for post‐OP. OP, operative.

The GRF analysis revealed no significant differences between MA and KA, either preoperatively or post‐operatively. However, a pronounced difference was observed compared to the healthy control group, with markedly reduced peaks in the vertical GRF during the loading response and terminal stance phase. Additionally, the horizontal GRF peaks were lower in both alignment groups compared to the healthy controls (see Figure 3).

**Figure 4:**
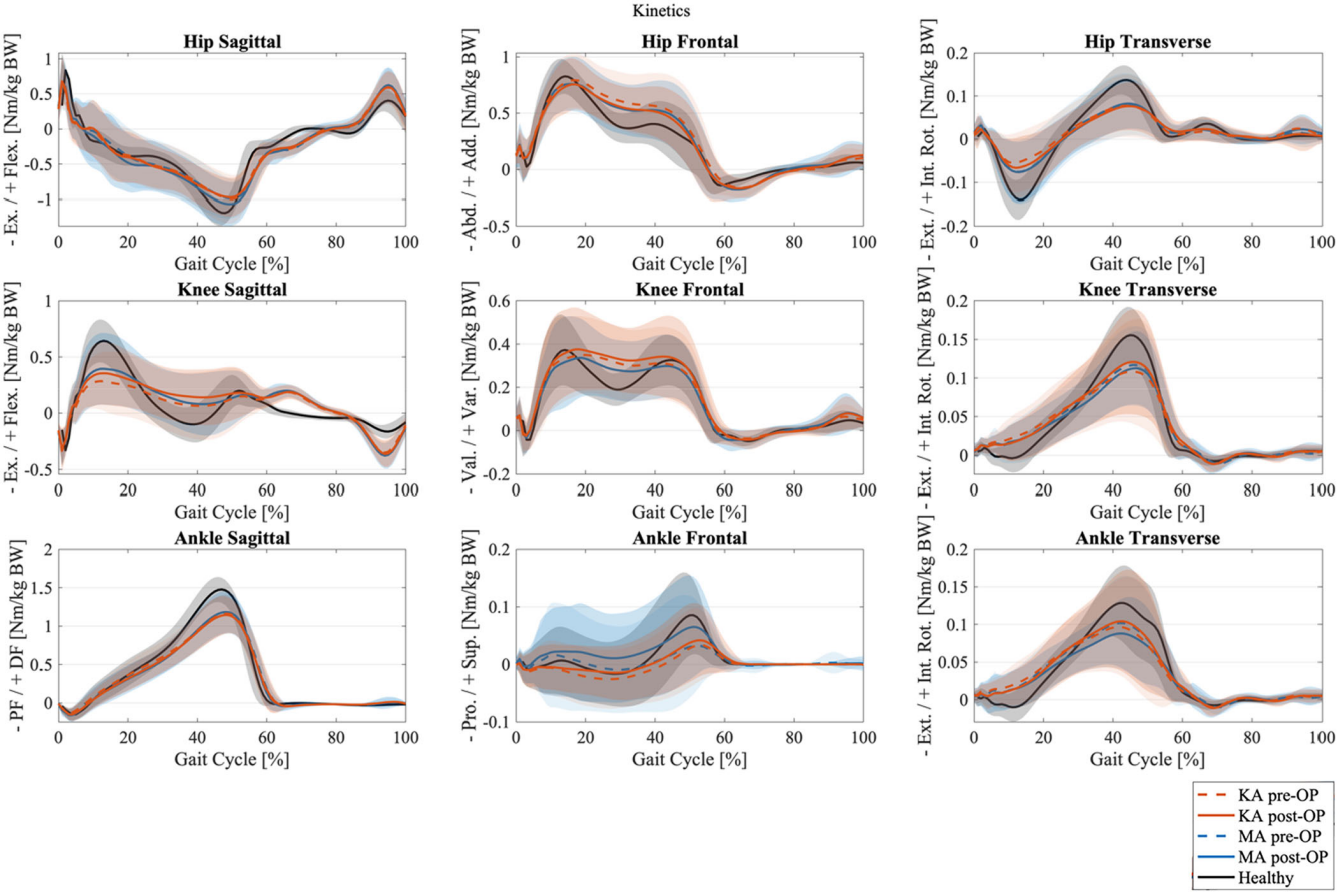
Kinetics for hip, knee and ankle joint in sagittal, frontal and transverse plane for kinematic alignment (orange), mechanical alignment (blue) and healthy controls (black) with mean and standard deviation; dashed line for pre‐OP and solid line for post‐OP. OP, operative.

The kinetic analysis using SPM showed no significant differences between KA and MA in any of the movement planes. In the sagittal plane, no differences were observed. In the frontal plane, no significant differences were detected; however, there was a tendency towards higher knee moments between 15% and 60% of the gait cycle, corresponding to the stance phase, for KA. Additionally, a high SD was observed. Similarly, in the transverse plane, no significant differences were found, but there was a tendency towards higher moments for KA, with both the knee and ankle showing a high SD (see Figure 4). The results of the SPM for the kinetics comparison of MA and KA post‐OP are in Supporting Information S1.

## DISCUSSION

The main finding of the study is that the modified KA does not produce different clinical outcomes compared to MA. Even among the numerous gait analysis parameters collected, no single parameter demonstrated any advantage or disadvantage of KA over MA.

Our study demonstrated that the tibial cut performed in the KA group resulted in a more varus orientation, while the femoral cut was positioned with greater valgus. This combination produced an overall post‐operative HKA angle of approximately 1.4° in the KA group. The intraoperative navigation data reflect the differing alignment principles in the positioning of the tibial component. Differences in femoral alignment were observed in many cases with KA but averaged out, resulting in no significant difference in the mean femoral component positioning between MA and KA.

One major difference, which simplifies the surgical procedure, is the frequency and extent of soft tissue releases. Here, KA shows an advantage, as the initially more symmetrical flexion and extension gaps substantially reduce the need for releases. This observation is consistent with other studies, which also report a lower frequency of soft tissue releases with KA [11]. These findings contradict current meta‐analyses that also examine a 1‐year follow‐up and report differences favouring KA [19]. Previous studies, such as those by Tian et al., have shown that KA is associated with fewer soft tissue releases [30]. Moreover, recent work by Shichman et al. suggested that the joint line orientation is less altered in KA and is better reconstructed [27], potentially contributing to improved functional outcomes.

With regard to PROMs, our results at 1 year after TKA did not reveal significant differences between KA and MA groups. The work of Dossett et al. reported significant improvements in the Oxford Knee Score and WOMAC in the KA group at a 2‐year follow‐up; however, it should be noted that in their study, KA was performed using patient‐specific guides while MA was executed with conventional instruments [8]. In our study, however, the radiological alignment parameters were comparable to those reported by Dossett et al., suggesting a convergence in post‐operative alignment despite differences in instrumentation. Similarly, Ettinger et al. reported no significant differences in WOMAC scores and objective components of the Combined Knee Society Score (CSS) at both 12 and 24 months, although a significant improvement in the functional subscore of the Knee Society Score (KSS Function) was observed at 24 months [10]. A review by Roussot et al. further highlighted that across studies, PROMs yield contradictory results [24], and a meta‐analysis by Liu et al. indicated that the benefits of KA appear more pronounced at 6‐month follow‐up, with a diminishing effect at 12 and 24 months [19]. Overall, the duration of follow‐up appears to be a critical factor influencing the reported outcomes.

Analysis of spatio‐temporal parameters in our study showed no significant differences between the KA and MA groups, which concurs with Yeo et al., who found no differences in cadence, stride length or gait speed [34, 35]. This further supports the notion that although there may be biomechanical alterations at the joint level, these do not necessarily translate into detectable differences in global gait parameters.

Regarding kinematics, our data did not reveal significant differences between the TKA groups; however, when compared with a healthy reference group, both TKA cohorts exhibited reduced knee flexion and increased knee valgus. Blakeney et al. reported that KA knees exhibit kinematics closer to those of healthy controls than MA knees. Nevertheless, in this particular study group, the KA group also demonstrated increased velocity, approaching the speed of the healthy group. This observation is of particular significance, given the established relationship between velocity and numerous parameters of gait. The KA group was superior to the MA group, especially in the swing phase, which, however, is less relevant for the load on the knee joint, while frontal plane behaviour exhibited a vertical shift (with MA showing more adduction and KA aligning closer to abduction) without clear evidence regarding the rotational alignment when compared to healthy knees [3]. In the present study, no significant differences were observed between the groups in any of the three rotational planes of the knee joint. Similarly, Young et al. reported no significant kinematic differences at the 24‐month follow‐up [7].

With regard to GRF, the present study found reduced peaks in the vertical and medial‐lateral directions. A study by Jones et al. comparing unicompartmental knee arthroplasty and TKA with healthy controls also showed that vertical GRF peaks were reduced in patients with arthroplasty [15]. This finding suggests that both alignment strategies in TKA are similarly affected in terms of GRF, with minimal difference observed between KA and MA groups.

The kinetic analysis of our study revealed no statistically significant differences between the groups. We observed higher knee adduction moments (KAMs) in both TKA groups compared to healthy controls and a slight tendency towards lower sagittal plane moments in the MA group during the second half of the stance phase. These findings are generally consistent with McNair et al., who also reported marginally reduced sagittal knee moments for the MA group in late stance. However, while this study has noted higher internal rotation moments with MA, our data did not support this finding [21]. In the frontal plane, there was a trend for lower KAMs in the MA group, which is in line with the notion that lower KAMs are beneficial; yet, these differences did not reach statistical significance. Young et al. similarly observed no significant differences in kinetic parameters at 24 months [7].

In summary, our findings contribute to the growing body of evidence suggesting that while radiological and intraoperative alignment parameters differ between KA and MA—with KA exhibiting more varus on the tibial side and more valgus on the femoral side—these differences do not necessarily translate into marked differences in PROMs, spatio‐temporal parameters, or kinetic and kinematic profiles at 1 year post‐operatively. However, evidence from long‐term and multi‐timepoint studies indicates that follow‐up duration may be critical in discerning subtle functional advantages of KA. Future studies should aim for longer follow‐up periods and more comprehensive biomechanical evaluations to clarify these trends further.

This study has several limitations that should be acknowledged. First, although 100 patients were initially included and randomized equally between the MA and KA groups, only 41 and 44 patients in the respective groups completed the 12‐month follow‐up. This dropout of 9 and 6 patients per group slightly exceeds the commonly accepted threshold of 10%, and the missing data could potentially introduce bias or affect the statistical power of the results. Second, gait kinematics were assessed using the Plug‐In Gait (PiG) marker model, which relies on skin‐mounted markers and optical motion capture. Such systems are inherently susceptible to soft tissue artefacts and errors resulting from inaccurate marker placement. However, all marker placements were performed by a single experienced examiner following a standardized protocol, which likely minimized variability. Despite this standardization, it must be noted that the PiG model captures external limb motion and does not directly reflect true skeletal kinematics. Therefore, some deviation between measured and actual joint motion may persist. Additionally, physical activity levels during the 12‐month post‐operative period were not monitored. Variations in individual activity levels may have influenced the clinical and functional outcomes, but could not be controlled for in this study. PROMs were collected via standardized questionnaires. As with any self‐reported data, there is potential variability in how thoroughly and accurately patients completed these forms, which may affect data quality. In addition, it should be noted that the technique used was a modified KA, which differs from other personalized alignment methods, which is why it offers limited comparability with results from classical KA.

Finally, while the study design as a randomized controlled trial provides a high level of evidence, it remains a single‐centre study, which may limit the generalizability of the findings to broader patient populations or other clinical settings.

## CONCLUSION

In this randomized controlled trial comparing KA and MA in TKA, no significant differences were observed between groups in terms of patient‐reported outcomes, gait kinematics, kinetics, or spatio‐temporal parameters at 1 year post‐operatively. While intraoperative and radiological alignment parameters differed—with KA showing more varus tibial and valgus femoral cuts—these variations did not translate into measurable clinical or functional advantages at the 1‐year mark. Our findings support the growing consensus that, in the short term, both alignment strategies yield comparable outcomes. Long‐term follow‐up and more detailed biomechanical analyses are warranted to determine whether KA offers functional advantages over time.

## AUTHOR CONTRIBUTIONS

**Georg Matziolis**: Conceptualization; methodology; data acquisition; Writing—review and editing; supervision. **Markus Heinecke**: Conceptualization; data acquisition; Writing—review and editing. **Frank Layher**: Methodology; data acquisition; formal analysis; investigation; Writing—review and editing. **Julia Kirschberg**: Data acquisition; Writing—review and editing. **Leandra Bauer**: Data analysis; formal analysis; investigation; Writing—original draft preparation. **Matthias Woiczinski**: Formal analysis; investigation; Writing—review and editing; supervision.

## CONFLICT OF INTEREST STATEMENT

The authors declare no conflicts of interest.

## ETHICS STATEMENT

Ethics Committee of Medical Faculty Friedrich‐Schiller‐University Jena (IRB no. 5060‐02/17).

## Supporting information

Figure S1. Violin plots for Delta Score (post‐operative minus pre‐operative) for Knee Society Score (KSS), knee and function evaluation, WOMAC; VAS frequency, VAS strength and post‐OP Score for FJS for Kinematic Alignment – KA (orange) and Mechanical Alignment – MA (blue); white dots indicate mean value.
Figure S2. Statistical Parametric Mapping (SPM) for unpaired *t* test (*p* = 0.05) for comparison of kinematics for hip, knee and ankle in sagittal, frontal and transverse planes for mechanical vs. kinematic alignment post‐operatively.
Figure S3. Statistical Parametric Mapping (SPM) for unpaired *t* test (*p* = 0.05) for comparison of kinetics for hip, knee and ankle in sagittal, frontal and transverse planes for mechanical vs. kinematic alignment post‐operatively.

## Data Availability

The data that support the findings of this study are available from the corresponding author upon reasonable request.
