# Supplementary material for: No differences in functional and clinical outcomes after rehabilitation between modified kinematic and mechanical alignment in total knee arthroplasty: A randomized controlled trial
Source: Knee Surg Sports Traumatol Arthrosc. 2025 Aug 19;34(2):596–607. doi: 10.1002/ksa.70004 (PMC12850575; doi:10.1002/ksa.70004)
Supplement: Supplementary file 1 — Figure S1. Violin plots for Delta Score (post‐operative minus pre‐operative) for Knee Society Score (KSS), knee and function evaluation, WOMAC; VAS frequency, VAS strength and post‐OP Score for FJS for Kinematic Alignment – KA (orange) and Mechanical Alignment – MA (blue); white dots indicate mean value. Figure S2. Statistical Parametric Mapping (SPM) for unpaired t test (p = 0.05) for comparison of kinematics for hip, knee and ankle in sagittal, frontal and transverse planes for mechanical vs. kinematic alignment post‐operatively. Figure S3. Statistical Parametric Mapping (SPM) for unpaired t test (p = 0.05) for comparison of kinetics for hip, knee and ankle in sagittal, frontal and transverse planes for mechanical vs. kinematic alignment post‐operatively. [file KSA-34-596-s001.docx]

Supplementary Information (SI)

Does Kinematic Alignment Improve Patient Outcomes in Total Knee Arthroplasty After Rehabilitation? A Prospective Randomized Study

| 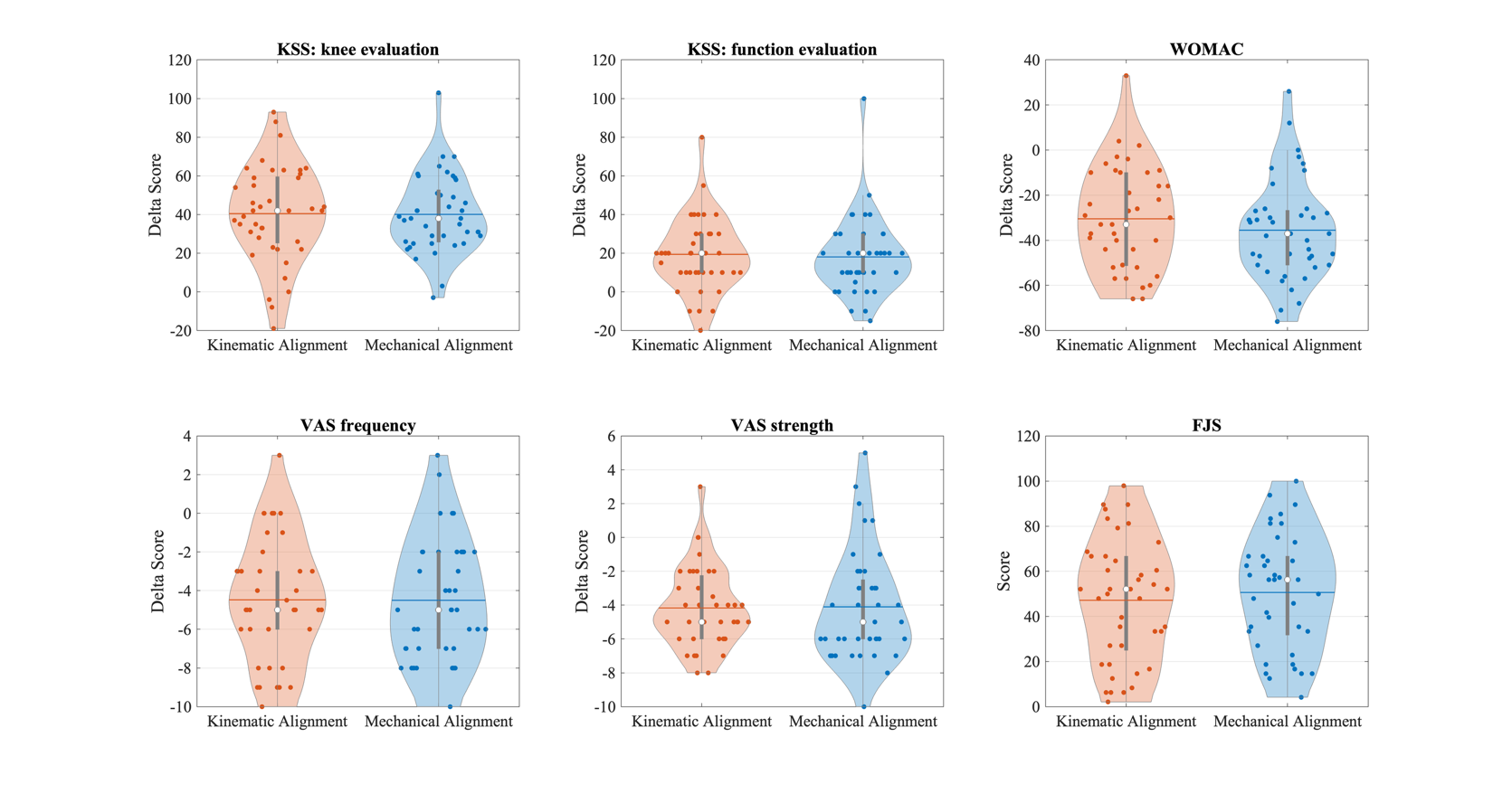 |
| --- |
| S. Figure 1: Violin plots for Delta Score (post-operative minus pre-operativ) for Knee Society Score (KSS), knee and function evaluation, WOMAC; VAS frequency, VAS strength and post-OP Score for FJS for Kinematic Alignment – KA (orange) and Mechanical Alignment – MA (blue); white dots indicate mean value. |


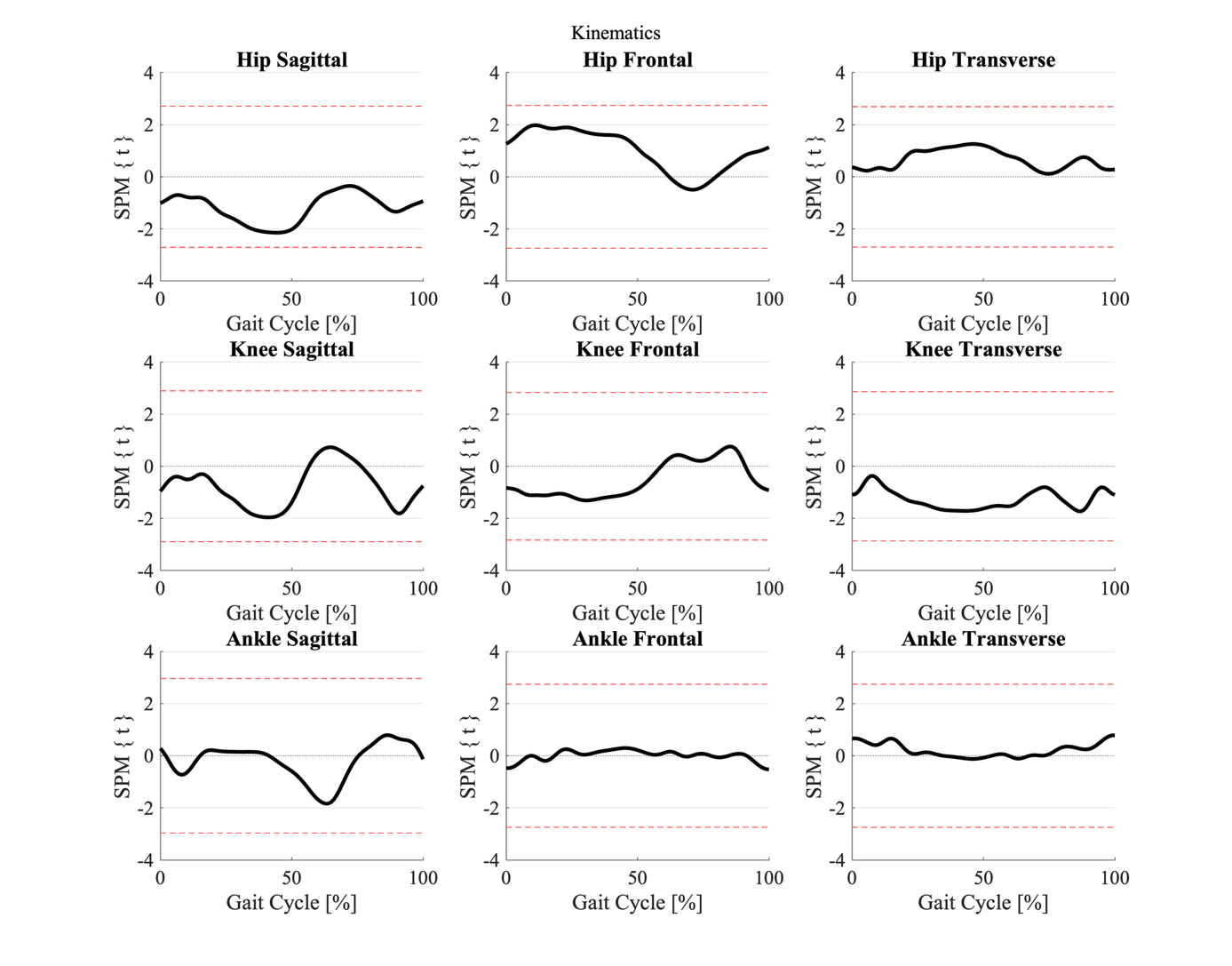


S.Figure 2: Statistical Parametric Mapping (SPM) for unpaired t-test (p = 0.05) for comparison of kinematics for hip, knee and ankle in sagittal, frontal and transverse plane for mechanical vs. kinematic alignment post-operatively.


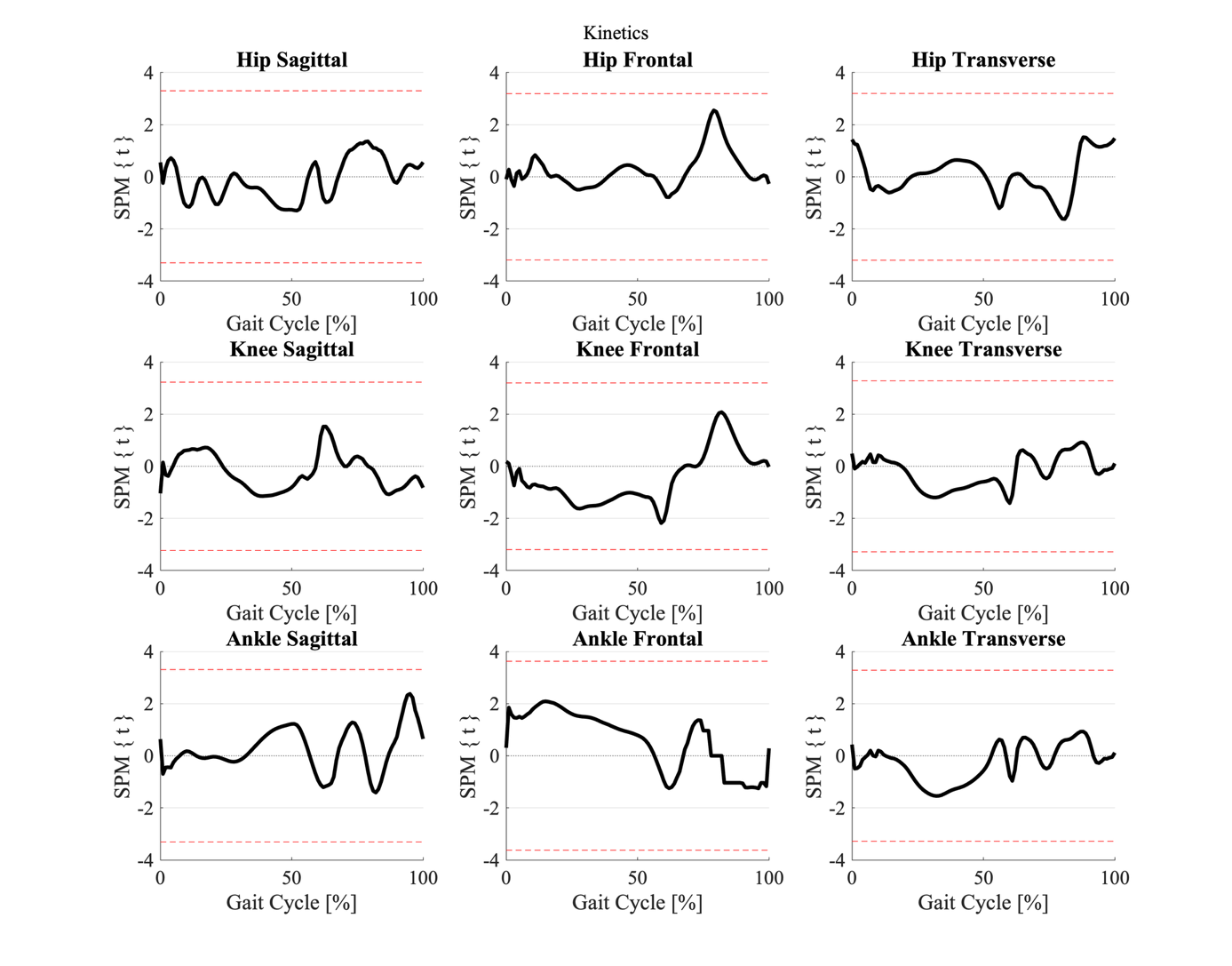


S.Figure 3: Statistical Parametric Mapping (SPM) for unpaired t-test (p = 0.05) for comparison of kinetics for hip, knee and ankle in sagittal, frontal and transverse plane for mechanical vs. kinematic alignment post-operatively.
